# Field robots for weed control? Analyzing socio-technical change by looking at farming practices

**DOI:** 10.1007/s11625-025-01739-y

**Published:** 2025-09-16

**Authors:** Anna Baatz, Katrien Van Poeck

**Affiliations:** 1 Center Technology and Society (ZTG), Technische Universität Berlin, Berlin, Germany; 2 Center for Sustainability Studies, Ghent University, Ghent, Belgium

**Keywords:** Field robots, Habits, Agricultural practices, Weed control, Socio-technical transition, Sustainability

## Abstract

The digitalization of agriculture is bringing about far-reaching socio-technical changes. This article analyzes these changes by looking at farming habits. Transactional theory of learning is introduced as an analytical perspective for investigating farmers’ consideration processes and experimentation with potential habit changes related to the use of digital technologies. The analytical perspective is applied to a case study of robotic weed control in sugar beet cultivation in northeastern Germany. The study shows how prevalent habits are crucial anchor points in farmers’ careful considerations of whether to use a field robot: habitual beliefs such as an excitement for robots, as well as professional and private habits and resulting free and committed capacities are included in these considerations. Experiences with technology use and weather-related uncertainties, furthermore, led to a habitual risk assessment and anticipatory solution seeking as an overarching element in the formation of new habits. In addition to the empirical study, the article aims to make a methodological and conceptual contribution to advance much-needed research on change of (agricultural) practices and the role of technologies in it. In this regard, the use of transactional theory of learning is discussed by reflecting on the kind of knowledge that can be produced with this type of analysis and how it can benefit research on practice change and broader (agricultural) transition processes.

## Introduction

The digitalization of agriculture is already changing farming practices (Annosi et al. 2024). Further extensive transformation processes in the context of digitalized agriculture are expected that will also shape potentials of transitions to more healthy and sustainable agri-food systems (Gugganig et al. 2023). This makes it highly relevant to investigate these socio-technical changes closely—in particular from a social science perspective (Klerkx et al. 2019). A crucial aspect of this is to analyze the (potential) use of novel technologies, the practices of adopting technologies, and the formation of novel routines that integrate the technologies (Schewe and Stuart 2015; Curry et al. 2021; Dissanayake et al. 2022). Various authors examine changes in agricultural practices, in terms of larger conversion processes with several entangled practices (such as the transition from conventional agriculture to organic) (Blackstock et al. 2010; Sutherland et al. 2012), as well as changes in individual practices such as the use of a particular technology (Schewe and Stuart 2015; Higgins et al. 2017). They thereby base their investigations on different theoretical frameworks and consider different factors and dynamics: (social) practice theory is used to conceptualize how routinized practices order the everyday life. Based on Giddens’ (1984) structuration theory, it considers cognitive and mental processes of actors (e.g., farmers) as well as more structural aspects such as regulations (e.g., Common Agricultural Policy), resources, material arrangements, etc. to explain (changes in) farming practices (Freyer and Bingen 2012; Jakku et al. 2019; Kaiser et al. 2024). A study by Higgins et al. (2017) argues that Actor Network Theory can function as a theoretical perspective to acknowledge “the specific ways in which aspects of the biophysical environment are weaved into how farmers engage with new technology” (p.198). Other authors are guided by Roger’s diffusion of innovation theory (1983), for example, or other frameworks that see certain trigger events as initial moments for change and define stages of practice change (Prager and Posthumus 2010; Sutherland et al. 2012; Chantre and Cardona 2014; Dumont et al. 2025). They show how a first stage can be the holding on to the status quo, followed by a trigger event that brings with it a desire for change, and afterward a step-by-step implementation of the change including the acquisition of further information. Individual factors (cognitive as well as affective/ emotional), and contextual factors (economic, institutional, environmental) play a role in the stages (Prager and Posthumus 2010; Sutherland et al. 2012; Chantre and Cardona 2014; Dumont et al. 2025).

In this paper, we introduce the transactional theory of learning as an analytical perspective on practice and habit change that can be useful to analyze the potential use of technologies. Its potential for analyzing habit change in the context of sustainability transitions has been argued conceptually and empirically (Van Poeck and Östman 2021; Baatz and Ehnert 2023; Baatz et al. 2024). On the one hand, this analytical perspective takes up several of the aspects of the above cited studies. This includes the consideration of individual and more structural aspects, attention for the interplay between people and their (social and material/non-human) environment, the crucial role of events in triggering change, and the understanding of change taking place in different stages or phases. On the other hand, it adds something to the above-mentioned theories and frameworks. It does so by offering an analytical lens that guides researchers’ views, enabling them to zoom in on (transformations of) habitual ways of thinking, acting, and engaging with the surrounding world (Östman et al. 2019; Van Poeck et al. 2020). Habits can thereby be understood as part of broader practices. Firstly, we argue that situating the analysis at the level of habits can help to study the so far underexplored temporal dimension of technology use practices (Dumont et al. 2025). This is because it allows us to trace how the habitual attitude toward a practice changes over time, even if the practice itself does not (yet) change. Transactional theory of learning is an analytical perspective to investigate trajectories of habit and practice change at different stages of change: when farmers are still undecided about the use of a technology, but also when they have already acquired new technology and are in the midst of incorporating it into their practices. The detailed empirical analysis of farming habits can, secondly, enhance the understanding of how farmers make up their practices. This type of analysis pays particular attention to moments in which habits are interrupted and subsequent consideration processes of the actors involved, to identify what (which attitudes, emotions, experiences, reasons, etc.) they take into account and how they do so in the formation of new practices. Elucidating these processes can reveal in depth how change of habits and practices unfolds over time, in action and thereby complement the analytical frameworks introduced above.

We apply this analytical perspective to a case study on the potential use of weed control robots in northeastern Germany. Farmers are currently gaining initial experience with the use of field robots (Spykman et al. 2023). However, it is still unclear whether and how farmers will use such robotic solutions (Schewe and Stuart 2015; Higgins et al. 2017; Jakku et al. 2019; Legun et al. 2023; Langer and Kühl 2023). This article therefore focuses on the integration of weed control robots into farming practices. We have a double aim. Empirically, we address two research foci: farmers’ considerations of integrating weed removal robots into their practices before farmers purchase a robot, and actual changes in routines after the purchase of a robot. We also have a conceptual–methodological interest of advancing research on agricultural practice change and the role of technologies. This applies in particular to the two aspects outlined above: enhancing the understanding of (1) the often non-linear, ongoing processes (temporal dimension), and (2) the detailed considerations and experimental habits involved in farmers’ practice change with the aim of integrating new technologies into their routines. Therefore, we also use the empirical work to test how the analytical perspective of transactional learning theory can be applied and help to understand how change comes about related to the use of new agricultural technologies.

In "Analytical perspective: transactional theory of learning", we outline the transactional theory of learning to study technology use and socio-technical change by focusing on habits. "Materials and methods: an explorative case study" summarizes the case study of sugar beet cultivation in northeastern Germany. "Results: analyzing the role of field robots in weed control habits" presents the results of the case study. In "Discussion and conclusion", we discuss the potential of the analytical approach for better understanding the use of new digital technology in agriculture, as well as its limitations and lines for further research.

## Analytical perspective: transactional theory of learning

Transactional theory of learning draws on the pragmatist philosophy of John Dewey (1934, 1938a) and focuses on actions and consequences. It has so far been used to analyze the interplay of learning processes and habit change in and out of school (Öhman and Östman 2007; Östman 2010; Van Poeck and Östman 2021). It is introduced here due to its analytical strength in analyzing the consideration processes and revealing the conditions under which actors change practices (Östman et al. 2019; Van Poeck et al. 2020).

Habits take central stage in the transactional theory of learning (Östman et al. 2019). As this article zooms in on *habits* to investigate the change of *practices*, it is important to clarify how both concepts are related, yet distinctive. While the meanings of the terms habits and practices resemble each other, especially in everyday language use, they have different roots (Volbers 2015): practices are the unit of analysis in practice theories, while pragmatists analyze habits. The transactional theory of learning is inspired by Dewey’s writings about habits. This theoretical conceptualization differs rather significantly from our mainstream, everyday understanding of the term. As Dewey (1938a, p. 35) emphasizes, his particular understanding of habits “goes deeper than the ordinary conception of *a* habit as a more or less fixed way of doing things, although it includes the latter as one of its special cases. It covers the formation of attitudes, attitudes that are emotional and intellectual; it covers our basic sensitivities and ways of meeting and responding to all the conditions which we meet in living.” (emphasis in original). We can thus describe ‘habits’ as habitual ways of doing, thinking, noticing, and feeling which we have acquired to gain “control over the environment, [the] power to utilize it for human purposes” (Dewey 1916, p. 52). Thus, for Dewey, habits cannot be reduced to mere routine modes of thought, observation, and behavior. On the contrary, he emphasizes “the intellectual element in a habit” which allows its “varied and elastic use” (Dewey 1916, p. 48) and provide us with active capacities to coordinate with evolving environments and readjust our actions in response to changing conditions. While practices are more overarching units in the everyday lives of actors, habits can be seen as more fine-grained habitual ways of doing, thinking, noticing, and feeling (Reckwitz 2002; Van Poeck and Östman 2021). An assemblage of habits makes up a practice. A person’s established ideas about robotic solutions, their way of perceiving amounts and location of weeds on the field, habitual ways of carrying out a small technical repair of a robot, and how one feels about working with human colleagues and robots, respectively, are all examples of habits that might co-constitute the practice of robotic weed control. Important to emphasize, considering the article’s focus on habits in relation to change of practices, is that from a Deweyan perspective, habits are not static abilities but temporary functions that are open to change as we learn from experience and transform established routines: “Plasticity or the power to learn from experience means the formation [and, we could add, transformation] of habits” (Dewey 1916, p. 52).

Focusing on habits, the transactional understanding directs the view to the interplay of actors and their environments: “People’s actions change the environment and shifts of activity happen in response to a changing environment.” (Van Poeck and Östman 2021, p. 158). Transaction here stands for an understanding of continuous interdependent relationships between people and their (human and non-human) environment. They transform continuously and reciprocally, *in transaction*, as one integrated system of co-existing organisms and environments separable only for analytic purposes (Dewey and Bentley 1949; Ryan 2011; for a detailed conceptualization refer to Garrison et al. 2022). It allows to examine simultaneous and reciprocal changes in actor–environment assemblages, and in particular habits and their consequences. Thereby, intrapersonal (such as previous experiences, values, knowledge, emotions), interpersonal (social interactions, power dynamics), institutional (traditions, discourses, policies), and material (vegetation, soil, infrastructure) aspects are considered (Van Poeck and Östman 2021).

**Fig. 1:**
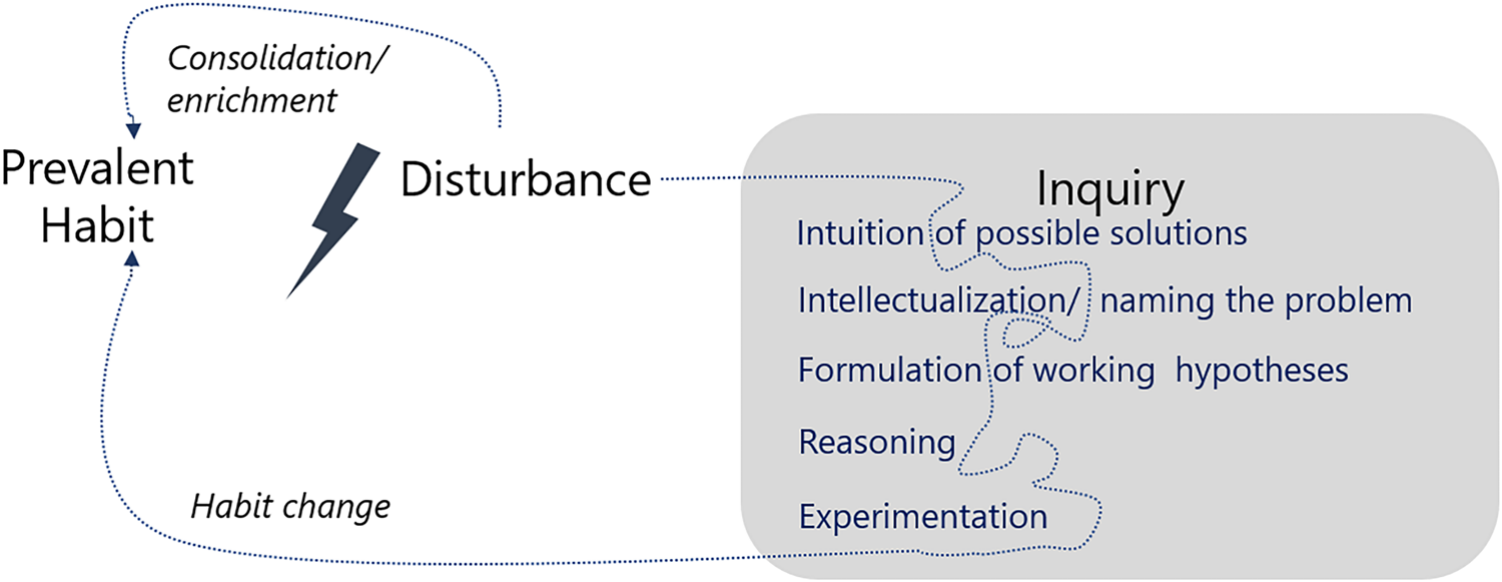
Transactional theory of learning (own illustration)

Once established, habits are relatively stable and not constantly reviewed. Sometimes, however, actors want or need to reorient their habits. This happens when prevailing habits are disturbed by (changes in) the surrounding conditions (as shown by Fig. 1). A disturbance causes an imbalance between the previous experiences, values, emotions, and something newly encountered that does not correspond to those and requires action to restore a functional way of engaging with the environment. Disruptions can be of different nature and occur on a cognitive but also affective level. Actors can deal with disturbances in different ways. They can cope by consolidating or slightly adapting existing habits. This resembles a quick return to the prevalent habit or a similar version of it. Actors can also deal with the disturbance more intensively. The non-reflexive mode of habits is then interrupted and replaced by an ‘inquiry’ into what is taken to be a ‘problematic situation’ (Östman et al. 2019, p. 131). Dewey (1933, 1938b) distinguishes the following phases in a process of inquiry: an intuition of possible solutions, intellectualization of the felt problematic situation into a named problem, formulation of working hypotheses, reasoning, and experimentation (see Östman et al. 2024 for more details). These phases can be overlapping and intermingled as the trajectory of inquiry is rarely linear and orderly. In the course of the inquiry, actors may take into account new knowledge, values, feelings, etc., and neglect some of the previously considered aspects. As a result, they might change their previous habit or develop a new one. Often an inquiry is not a distinct, isolated event arising from a specific disturbance in one situation, but, on the contrary, usually characterized by continuity and accumulation over time. Since “every experience both takes up something from those which have gone before and modifies in some way the quality of those which come after” (Dewey 1938b, a, p. 35), (trans)formation of habits is based on several disturbances or repetitions of disturbances. Farmers may, for instance, receive information about robotic weed control several times, combine this with their feelings of exhaustion from manual weeding, and only as a result of all these things start an inquiry to find out if field robots might be of interest to them.

In this paper, we investigate agricultural habits of weed control from a transactional pragmatist perspective. Farmers establish(ed) farming and weed control habits, based on intrapersonal, interpersonal, institutional, and material aspects. We trace their inquiries and focus on their processes of consideration and experimentation. Our first analytical focus concerns the consideration processes regarding robotic weed control. Here, farmers take an anticipatory perspective while thinking about how their farming habits could be organized if they would use a robot. The second analytical focus is on farmers’ experimentation with field robots, i.e., the reactive processes of farmers after the purchase of the technology.

## Materials and methods: an explorative case study

### The case: weed control in sugar beet cultivation in northeastern Germany

We present sugar beet cultivation and in particular (robotic) weed control in northeastern Germany as an interesting case to study habit change in relation to new agricultural technologies. The case study is rooted in northeastern Germany, including the federal states of Mecklenburg–Western Pomerania and Brandenburg. The region is characterized by large farms, sometimes with several thousand hectares. The region is home to an established sugar processing company, which aims to establish organic sugar beet processing and is looking for organic farmers to grow sugar beet. In general, (organic) sugar beet cultivation can achieve high profit margins. Timely weed control is a very critical factor. The potentially high profit margins in sugar beet cultivation and the associated financial scope allow for the introduction of new technologies. Farmers are already looking for new strategies to fight weeds, as it has become more difficult to find workers for manual weed control. This point of departure makes the case an interesting one to study: some farmers already grow organic beets, others would like to do so. Several farmers are interested in robotic weed control, but it is not yet widespread. This allows us to observe farmers consideration process of whether to include a robot in the weed control habits, as well as the first adaptions of habits due to the use of a field robot. The regional focus of the case ensures similar conditions in terms of the natural environment, policy regulations, as well as the marketing of beet.

In this article, two robots for weed control are examined: the Uckerbot (UB), which is currently under development, and the Farmdroid (FD) robot, which is already in use. The first author of this article is involved in the Uckerbots project, a transdisciplinary collaboration for technology development and implementation. A prototype of the UB emerged from a previous project. It is a small machine compared to other agricultural robots, equipped with a camera and linked to an artificial intelligence device. Image recognition is used to distinguish sugar beet from weeds, and the robot can then remove the weeds with various chopping tools. The name *Ucker*bot alludes to the *Ucker*mark region located in northeastern Germany. The FD robot works on a different principle. It uses GPS data. The robot sows the sugar beet plants and memorizes their position. Any plant growing elsewhere is then weeded out. There are advantages to the principle of precise positioning. For example, it is possible to weed very early, even before the sugar beet plants emerge. However, farmers also mention that the FD struggles to drive on uneven ground, which is very common in the region. Also, weeds that grow close to the sugar beet are not weeded due to the safety distance (Steinherr et al. 2023). The FD is currently the most widely used field robot in sugar beet cultivation (Spykman et al. 2023).

### Case study design

We chose a case study design to gain an in-depth understanding of weed control habits (Yin 2008, 2014). The study of disturbances is complicated by the fact that they occur at unpredictable moments. Several disturbances can also follow one another over a long period of time. We therefore decided to ask farmers to reflect in semi-structured, qualitative interviews on their current farming habits, disruptions, inquiries, and potential habit changes related to the use of weed control robots. Five farmers, an agricultural expert who advises farmers, and a person from a local business development office were interviewed (*n* = 7). The interviews were conducted online, recorded, and transcribed. A limitation of the procedure is that the data have a sample bias. Only farmers with at least some interest in field robots responded to the interview invitation. Due to the novelty of field robots, there are only a small number of users in the northeast of Germany. The bias and the explorative character of the research in a novel field also led to a comparatively small number of interview partners. While the farmers all describe their relatively different farm structures, there is nevertheless a certain degree of data saturation with regard to their consideration processes.

To compensate for the small number of interviews and to observe less conscious processes that cannot be queried in interviews, participatory observations were conducted and captured by fieldnotes (*n* = 12, see Appendix 2). Field notes were taken at various events and occasions, for example, during a field day on an organic farm. Approximately 12 farmers (including 2 farmers who were also interviewed) and 4 advisors met and new weed control technologies such as the UB and the FD were presented. Farmers using or testing the technology provided progress reports. Other field notes stemmed from a UB project meeting in which farmers reported on their experiences with testing the UB. The first author took the field notes, paying particular attention to the farmers' reactions to the demonstrated field robots, as well as their questions and discussions. These observations of direct reactions and interactions complement the interview data.

We analyzed the data following an interpretative approach, combining deductive and inductive coding (Kuckartz and Rädiker 2022, p. 103f). Transactional theory of learning and its focus on actions and consequences served as a starting point. Accordingly, in a first step, passages referring to habits were identified in the interviews and field notes. We distinguished between habits of sugar beet farming in general and weed control habits in particular. More general passages on sugar beet cultivation tend to contribute to the case description (in 3.1), while passages specifically on weed removal form the core of the empirical material to be further analyzed.

In a second step, the coded material was interpreted more intensively. We searched for comments, arguments, or descriptions of habit disturbances, inquiries, and habit changes related to the use of field robots. Table 1 summarizes these concepts and breaks them down for empirical application. This should help to understand the analytical process of interpreting the material. The distinguished intrapersonal, interpersonal, institutional, and material aspects are not explicitly operationalized, but serve as a sensitizing concept, in the sense that they sensitize us to consider all these different aspects in the analysis, should they be raised by the farmers. Thus, they consistently help focusing our analytical attentiveness on the ‘transactional’ perspective on change-making (i.e., the continuous, dynamic interplay between those aspects) that underpins our theoretical framework. In contrast to the operationalized concepts, however, they are not used to structure the analysis.

**Table 1:** Operationalization of concept for data analysis

Concept	Description and operationalization for data analysis
Habit	A farmer describes a habitual way of doing, thinking, noticing, and feeling by describing things they are used to do, things they take for granted, and established beliefs and values. Sometimes, these habits are explicitly mentioned by farmers in observed activities. At other occasions, however, we can identify them by observing what ‘stands fast’ (Wickman and Östman 2002) for the farmers, that is, what they take for granted and remains unquestioned, thereby implicitly reflecting a habit
Disturbance and problematic situation	Something catches the attention of a farmer and interrupts the non-reflexive mode of habits. In the interview data, we mostly could not observe in situ how farming habits were disturbed, but had to look for comments/reports referring to surprising moments, new feelings, thoughts, and reflections (i.e., where the disturbance was taken as a problematic situation). We identified references to things that used to stand fast (habits) and that are now questioned or caused difficulties to (know how to) proceed with one’s usual practices because of the disruption
Inquiry	The farmer tries to deal with the problematic situation through reflection and experimentation. The farmer may reflect on and question the established farming habits, possibly consider new information, meanings, emotions, and (virtually) try out different habits. Five ideal–typical phases of inquiries that can occur in different orders and may be discussed in interviews
1) An intuition of possible solutions	Farmers develop an initial sense of where to look for possible solutions. This is one of the ideal–typical aspects in an inquiry that is less likely to come up in interviews as we are not observing in situ and it might not be recalled retrospectively
2) Intellectualization/naming the problem	Farmers specifically pinpoint and formulate the problem. This facilitates further inquiry
3) Working hypothesis	Farmers formulate a guiding idea to solve the inquiry that needs to be tested
4) Reasoning	Farmers imagine the consequences of acting upon the guiding idea (dramatic rehearsal^a^)
5) Experimenting	Farmers describe how they test the hypothesis by overt action

^a^Dewey (1908, p. 293) describes such “imaginative rehearsal of various courses of conduct” as follows: “We give way, *in our mind*, to some impulse; we try, *in our mind*, some plan. Following its career through various steps, we find ourselves in imagination in the presence of the consequences that would follow; and as we then like and approve, or dislike and disapprove, these consequences, we find the original impulse or plan good or bad”

Having elaborated the theoretical background of our study, we can now specify our research questions for the case study as follows:

- When do farmers consider robotic weed control? What disturbs their habits?
- What do their consideration processes include? How do inquiries proceed?
- How does the integration of a weeding robot affect changes in farming habits?
- Which consideration processes and inquiries took place after the purchase of the robot?

## Results: analyzing the role of field robots in weed control habits

The presentation of the results starts with the consideration processes of farmers and the anticipatory aspects of their inquiries (“Integration of robotic weed control in current farming habits”). “Adapting cultivation habits to a weeding robot” deals with inquiries related to how farmers adapted and changed their habits (reactively) after introducing a robot to the farm.

### Integration of robotic weed control in current farming habits

All five interviewed farmers expressed their interest in growing organic sugar beet. Two of them are already growing sugar beet using a FD robot for weed control. They consider supplementing the FD robot with a UB, so that the UB hoes the weeds that grow close to the sugar beet plants and are not removed by the FD.

During the interviews, the farmers talked about multiple cultivation habits that were disturbed. Several of these disturbances were related to the overall challenge of lacking reliable weed control strategies. In the past, weeding was habitually organized by employing seasonal workers. However, the environment for this habit changed as it has become difficult to find reliable workers and to provide accommodation for them, especially in tourist areas; also, labor costs have risen (I4,5,6).

The development of field robots was a starting point for the interviewed farmers to start or reactivate their ongoing inquiry regarding finding a reliable weed control strategy. In the following, we outline how different farmers start an inquiry on robotized weed control. These inquiries often refer to their current farming habits, what they usually do. Table 2 summarizes the given examples by specifying the habits which the farmers addressed, the disturbance, and the aspects brought up in their inquiry.

**Table 2:** Summary of results regarding the adoption of robotic weed control habits

Interview number	Prevalent habits addressed	Disturbances	Inquiry and related change of habitual ways of thinking, acting, noticing, and feeling
I2	No organic cultivation with the available technology	Seeing the FD in a magazine	*Intuition of possible solutions*: Use the FD
	Not feeling like employers		
	Wish to not bring in a labor force from Eastern Europe		
	Interest in development of robotics		
I5	Need to remove remaining weeds after FD use by hand	Manual work is monotonous	*Working hypothesis*: a robot could do the job
I6	Concentrated workload peaks	The requirement of robot supervision	*Reasoning*: the farmer cannot supervise the robot *Working hypothesis*: using a contractor with a weed-bot
I2	More time as children have grown up	Question about current farming strategies	*Reasoning*: own supervision of the robot *Working hypothesis*: this fits the work structure
I5	Uncertainties about weather conditions, technological malfunctions, etc., and related need for risk control	Question on the conditions for robot use	*Working hypotheses*: wish to test/rent the robot before buying
FN1, P11	Uncertainties about weather conditions, technological malfunctions, etc. and related need for risk control	Observing a technology demonstration of the UB	*Reasoning:* the low working speed could be problematic because often windows for weed control are short
I4	Uncertainties about weather conditions, technological malfunctions, etc., and related need for risk control	Questions on required support services to use the robot	*Working hypothesis*: spare parts have to be delivered the next day
I5	Positive experiences with solving technical problems of the FD	–	*Working hypothesis*: it would be similarly possible with the UB to receive feedback via a hotline as everything is accessible online
I5	Experience with the need for risk control in relation to pests and diseases	–	*Working hypothesis*: UB’s image recognition technology can be used to detect diseases or pests at an early stage

While the disturbances of habits took place before the interview, farmer Carla^1^ recalls her experiences in detail: *We had actually given up growing sugar beet. Organic cultivation with the available technology was not an option for us. Neither was manual labor, given our farm structure, or rather a personal, emotional decision. We simply don't feel like employers, and certainly not in the context of bringing in a labor force from Eastern Europe. That is not for us. We've always waited for the right robot to finally come on the market. […] We were always on the lookout, and then we saw the Farmdroid in a magazine and immediately knew it was the robot for us, even before we had seen it live"* (I2). She outlines how in the past she started an inquiry to find a solution for weed control, and in the course of reasoning considered hiring workers to weed manually, but excluded this option inter alia because of emotional, affective aspects. Accordingly, Carla formed the new habit of not growing sugar beet. Noticing the availability of field robots in a magazine disturbed this habit. In another part of the interview, she describes how the habit of a long-lasting interest in agricultural robots and the belief that they will play a major role in future cultivation strategies guided her inquiry and facilitated the emergence of the working hypothesis that the Farmdroid robot can solve their problem.

In their inquiries, the farmers think about how robotic weed control could work and which tasks the robot should take over. Farmer Julius does so by referring to his habits of using the FD and remaining weeds: “*The FD robot manages to remove 90% of the weeds and we have to remove the remaining 10% by hand. And the manual work is actually so monotonous that, in my opinion, a robot could do the same job.*” (I5). His inquiry regarding robotic weed control is based on the embodied experience of monotonous manual weeding work. In this respect, the working hypotheses of the interviewed farmers are similar as they wish to replace the hard, manual weeding work and consider the use of a robot as a solution (I2-6, P9).

Another consideration refers to newly arising tasks such as training and monitoring the robot. Encountering the requirement of robot supervision functions as a disturbance. Farmers deal with this disturbance in varied ways, related to varied existing habits. Dairy farmer Johan reflects on the problem of peaks in workload during the weeding time window when imagining the consequences of having to train and supervise a robot: *“I can't do it at that time. I have silage at the end of May for my cows. We have a lot of work peaks, and they all come on top of each other. Like this year, when sowing took place three weeks later, the workload is concentrated at peak times. So, there's more and more work and the time frame is getting shorter and shorter.”* (I6). Reasoning about the supervision of the robot in relation to his diverse habits cumulating in workload peaks, Johan comes up with a new working hypothesis: “*If a contractor would do this with a weed-bot, especially when a dairy farm already has to deal with maize hoeing anyway, then that would of course be the top option. That's a good idea.”* (I6). The temporal shifts in habits, such as the three-week delay in sowing, are also related to weather conditions, which are discussed at the end of the section.

In contrast, Carla refers to freed-up capacities in childcare and thus comes to the working hypothesis that she can take over the care of the robot herself: *“Our children are grown up. Two have already moved out and the little one is twelve. Time has become free. So, I'm there for the robot from March to June. This fits in quite well with our work structure.”* (I2). This demonstrates how existing habits (here: childcare habits) are crucial anchor points in inquiries and decisive for the reasoning and the establishing of working hypotheses.

Furthermore, farmers frequently refer to uncertainties and the necessity for risk control. They indicate that their cultivation practices are already characterized by several uncertainties related to temperature development in spring, rainfall or pests. Weed control habits depend in particular on soil moisture and dryness: “*And we are in agriculture, we work with the weather, and it can happen that you stand there for a week and think, yes, I should be out in the field every morning and keep trying, but I can't because it's too wet or because this is or that.*” (I1). These weather-related, material aspects and the uncertainties associated with them play a central role in the reasoning processes. Farmers engage in a dramatic rehearsal, a process of imagining how they could deal with different disturbances due to weather conditions (such as moist soil) or technical malfunctions when using a weed control robot. They habitually aim to assess possible risks and problems, to anticipate potential future disturbances that could arise, and to develop possible strategies (I6, I4).

On the field day, farmers joined a technology demonstration of the FB and the UB. The demonstration served as a disturbance for some famers, who observed the robots closely and imagined using it (FN1, P11). They were more skeptical than the farmers interviewed. Five of the present farmers discussed the fact that the periods for weed removal are very short in some years due to the weather and in this context problematized the use of the UB as they perceived its working speed as very slow. They came to the working hypotheses that they currently do not plan to use the field robot because of this.

The farmers interviewed are habitually well aware of problems that new technologies can have, and accordingly their reasoning results in several strategies for reducing the uncertainties associated with using the robot. Therefore, Julius answers the question under what conditions he would use a robot by referring to the purchase model: *“I do not want to lease the robot. Renting would be an option. Or testing it for a week and writing a report on what's going well and what needs to be improved. And then I'll know whether I want to buy it next year or not.” (I5*).

In a similar way, a question on the required support services triggers a reflection. Farmer Christian anticipates malfunctions of the robot and includes them in the reasoning. As a result, he formulates a condition for robotic weed control habits: *"Spare parts have to be there the next day. The chopping window is only four weeks, and if it rains, you have only three weeks in the very critical phase of sugar beet." (I4)* Julius refers to positive experiences with solving technical problems of the FD (already established habits) and formulates a working hypothesis that it will be probably similar for new weed control habits using the UB: *“You can do quite a lot yourself on the equipment [of the FD robot] and everything else can be done over the phone. It worked quite well with the FD and I think it would be similar with the UB, if you have problems, everything is online accessible.” (I5).*

All farmers imagine potential problems and come up with risk control strategies, several distinctive habits of dealing with insecurities, and corresponding requirements for the robot and its business model. Based on their experiences with the shortness of the weeding window (habitual belief), they request that support services need to be constantly available during the respective weeks (I1, I2, I4, I5). They thereby envision and propose new habits along the sales of the robots. While most of these inquiries dealt with reducing the risks of using the UB, one farmer’s reasoning resulted in a working hypothesis on how the UB could be further developed in a way that can potentially reduce risks. He draws on existing habits (in particular, the experiences with diseases) and, encountering the UB’s image recognition technology, comes up with the creative suggestion to implement an additional function of interpreting the collected visual data to detect diseases or pests at an early stage (I5).

### Adapting cultivation habits to a weeding robot

The second part of the results summarizes how farmers transact with the robot, by using it and adjusting their habits to the robot (summarized in Table 3). As the UB has not yet been launched, the results are based on interviews with two farmers who work with the FD, and with the cultivation expert.

**Table 3:** Summary of results regarding habit changes after starting to use a robot

Interview number	Prevalent habits addressed	Disturbances	Inquiry and related change of habitual ways of thinking, acting, noticing, and feeling
I2	Not growing sugar beet	Availability of weeding robot	*Experimenting*: testing the robotic weed control in rapeseed and then starting in sugar beet cultivation
I2	Anticipation that problems might arise with robotic weed control	–	Manual weeding to supervise the robot while it works
I2	Starting with sugar beet cultivation using robotic weed control	FD collides with hunting stand	Acknowledging safety distance and sowing a strip of oat to ensure the safety distance
I5	Starting with sugar beet cultivation using robotic weed control	Noticing supervision requirements at least twice a day	Using the robot close to the farm or to the employees' homes
I2	Starting with sugar beet cultivation using robotic weed control	Robots’ problems to drive on uneven grounds	Only choosing flat fields for the FD
I2	Requirements to set aside land or to sow flower strips		Sowing a flower mixture where there is too much slope for the FD

The two farmers, Carla and Julius, report that they only started sugar beet cultivation because the FD offered a chance to remove weeds with little manual labor (I2, I5). The decision to use the FD was an attempt to overcome the problematic situation of lacking reliable weed control strategies and to establish new weed control habits. After the delivery of the FD in autumn, Carla has carried out a test cultivation to practice the use of the machine (I2). Experimentation to learn how to use the FD includes the first transactions with the robot and can be interpreted as part of an inquiry on robotic weed control: “*In autumn, we planted 6 hectares of rapeseed to test the robotic weed control. In the spring of 2022, we started with 20 hectares of sugar beet because we already had the experience with the machine." (I2).*

She describes her new habits of sugar beet cultivation in the subsequent season: *“I chopped what I could myself. […] Then I'm practically outside with the robot and I'm always there immediately if something happens. […] So at the beginning we were negatively surprised about the required safety distance. […] At one point it [the FD] knocked over a hunting stand. After that we learned that we just have to react differently. This year, for example, we have sown a wide strip of oats all around the field.” (I2).* After buying the robot, she developed the new habit of manual weeding while the robot also weeds. The new habit relies on the anticipation that problems might arise and her wish to be directly on site. Such a problem she could observe was a collision of the robot with an obstacle at the edge of the field. This disturbed her weed control habits. In the course of an inquiry, she developed the new habit of sowing oats around the field as a buffer zone to maintain a safety distance.

Julius describes similar adaptions of his farming practices after introducing the robot: *"We have 200 hectares here and we use the robot here and not on fields 10 or 15 km away, because I've found that I have to go there once in the morning and once in the evening and there's a control effort involved. Of course, you have to take that into account." (I5)* Here, he refers to his experience of having to monitor the robot regularly. In the past, disturbances arose as malfunctions of the robot or in form of the need to reset and adjust the robot. These disturbances informed further habits of robotic weed control in the following seasons.

Carla further introduces another new habit: *"We only chose flat fields for the Farmdroid. We actually had it in the rape in autumn. It was wet and I went there quite often and pushed the robot up the hill. […] Now we manage it with crop rotation that we only sow sugar beet on flat land. Also, we have so many requirements now that we have to set aside land or have to sow flower strips or something. We've already said, okay, we will just have to measure out a corner and somehow sow a flower mixture there, and then it [the FD] just has to drive around a hectare or something where there's too much slope. So, with experience of using a robot you become flexible, you just have to know that beforehand." (I2).* The robot’s problem with driving on slopes disturbed her weed control habits. She reacted in two ways. In the course of her inquiry, she plans to sow beet only on level ground that the FD can drive on and thereby established a new habit. Furthermore, she added something to this strategy by including the habit of sowing flower mixtures to increase biodiversity. The creative solution to both (the need for sowing flowers and the problems of using the FD on uneven grounds) is to plant the flower mixtures on the uneven parts of the sugar beet fields. In this way, she forms a new habit in response to the inquiry and by including an existing habit.

## Discussion and conclusion

This article has a twofold aim: (1) to create empirically grounded knowledge about farmers’ habit changes related to the use of weed removal robots, and (2) to test the potentials and limitations of the transactional learning theory for progressing our insight into how practice change comes about related to the use of new technologies. This section first discusses the produced knowledge and the potential of the employed analytical approach to shed new light on the acceptance and use of new digital technology in agriculture (“Gaining insight into the use of digital technologies and agricultural habit and practice change”). Subsequently, we discuss the limitations of the analytical perspective of transactional theory of learning (“Limitations and needs for future research”).

### Gaining insight into the use of digital technologies and agricultural habit and practice change

How do the empirical insights from our case study confirm, contradict, and complement earlier research on the use of new digital technologies and practice change in agriculture? By discussing this, we outline the strengths of the presented analytical approach and its potential to deliver novel insights complementary to the knowledge created with other, often employed approaches. As we explain and illustrate below, an analytical lens focused on the change of habits allows to open the black box of technology adoption and practice change at a fine-grained level and to create practically useful knowledge. Table 4 summarizes the gained insights and strengths of the analytical perspective we introduced.

**Table 4:** Summary of gained insights

Findings from previous studies	Our insights	Explanation on how this adds to the existing knowledge/ enhances the analytical perspective
Economic aspects and reliability of the technology are central for adoption (von Veltheim and Heise 2020; Spykman et al. 2021)	Our findings support this and add to it: we found that weather conditions often lead to a short time window for weeding. Therefore, farmers aim to ensure that the robot is ready for use by developing risk control strategies	We confirm previous findings, adding details on why and how they matter. The analysis shows that farmers interlink intrapersonal, material and institutional aspects
Farmers have privacy concerns regarding precision agriculture technologies, they also fear to lose local connectedness (Jakku et al. 2019; Janc et al. 2019)	These aspects were not part of the inquiries of the farmers in our sample	We cannot confirm these aspects. It might be because these (existing) concerns do not become part of the inquiries we analyzed. This needs further research focused on habits and practices
–	Linkages between practices (e.g., farming and child rearing) affect the development of new (weed control) practices. Prevalent habits are crucial anchor points in the considerations of using field robots. Farmers habitually assess risks and seek solutions in anticipation. The examples also demonstrate creative and flexible actions of integrating the field robots into farming routines	In addition to the so far discussed findings, we outline how entangled practices affect practice change. We further identify certain patterns in inquiries, ways in which farmers consider technology use, and stress the plasticity of habits
Change processes can be described by defining different stages (Prager and Posthumus 2010; Sutherland et al. 2012; Chantre and Cardona 2014; Dumont et al. 2025)	We identify different disturbances and inquiries that sometimes result in habit and practice change and sometimes do not. We show how farmers sometimes wait for conditions to change and then continue previous inquiries	Describing different disturbances concretizes how habit change can be initiated. Tracing the inquiries over a period of time emphasizes the temporal dimension, the non-linearity of habit and practice change
Relevance of individual and more structural aspects for practice change (Prager and Posthumus 2010; Chantre and Cardona 2014; Schewe and Stuart 2015)	In inquiries, farmers refer to intrapersonal, interpersonal, institutional, and material aspects, and in particular to their interplay	The focus on inquiries allows to consider intrapersonal, interpersonal, institutional, and material aspects without assuming their relevance. They rather serve as sensitizing concepts. This is a strength of the introduced analytical perspective compared to previous studies
–	We describe changes of habits as prerequisites for potential practice change	This can help to better understand how change comes about in action

Findings on the use of new digital agricultural technologies come mainly from acceptance and adoption studies (Jakku et al. 2019; Janc et al. 2019; von Veltheim and Heise 2020; Spykman et al. 2021). They point to the relevance of economic aspects, reliability, privacy concerns, and a fear of losing a local connectedness for the adoption of digital agricultural technologies (such as field robots) (ibid.). In the following, we show how our analytical perspective can complement these findings. Due to the small data sample, we limit ourselves to showing what kind of knowledge can be produced by this type of analysis.

The farmers in our study were also concerned with the economic aspects and reliability, and our findings show how far these mattered in their inquiries. As the time window for weed control is short, attempting a new strategy involves risks relating to technological malfunctions and weather conditions. In their consideration processes, farmers aim to reduce these risks by having ready-made strategies to deal with problems. This implies that a business model for field robots needs to include the option to test the robots prior to purchase, as well as trainings, and support services in the ,event of technical malfunctions. The above presented analyses illustrate how a transactional analytical approach allows to gain detailed insight into how farmers interweave intrapersonal aspects such as experiences with new technologies with material aspects (weather conditions) and come up with institutional requirements (support services) for using the robots.

The farmers interviewed were not concerned about data protection, nor did they fear losing their connection with their local community. One farmer even requested further analysis of the UB's collected data. The differences between the results of this study and previous research can be partly explained by the sample bias of this study. They may also be due to the fact that, unlike the technologies analyzed in some of the previous studies, the UB is being developed by a small company in collaboration with a university, rather than by a large multinational company. This could create trust. However, it could also be that concerns about privacy and local affiliation exist, but farmers do not consider them in their inquiries and habit formation. This needs further research, particularly with a view on habit and practice formation.

In addition, the conducted study guided by the transactional theory of learning points to novel aspects relevant to the decision to use a robot for weed control that have not been described before. It revealed how various habits—ranging from established beliefs about robotics to bodily experiences of hard manual work—played a crucial role in farmers’ inquiries. The case study revealed, for instance, how professional and private habits were mentioned in connection with free or committed capacities for robot supervision. These include the cultivation of specific crops, the keeping of livestock, corresponding capacities of seasonal workers employed, and family habits such as child rearing. Here, too, it is the transactional focus on the interplay between intrapersonal, interpersonal, institutional, and material aspects that helps gaining detailed insight in such dynamics. Through the analyses, also the entanglements of habits become visible. Similarly, practice theoretical perspectives highlight how actors link practices with one another creating entanglements (Pantzar and Shove 2010; Baatz 2024). The findings show that—based on different prevalent habits—farmers have different ideas of the robotic weed control, e.g., preferring to use the robots as a service via a contractor or supervising the robot themselves. This complements the findings from Schewe and Stuart (2015) that adoption of milking robots is manifold depending on the personalities of farmers and farm structures. The finding entails implications for political programs promoting digital agricultural technology use and related business models. These should develop different offers tailored to different farm structures and habits.

We also observed how prevalent habits are important anchor points for future habits, e.g., experiences from working with the FD robot inform inquiries about the use of the Uckerbot. Farmers engaged in a dramatic rehearsal of potential problems and ways to solve them. They rely on their habitual risk assessment and anticipatory solution seeking, which seem to have arisen from accumulated earlier experiences with challenging weather conditions, technical malfunctions of new technologies, and the interplay of both. Revealing this overarching element in the formation of new habits sheds new light on the role of (potential) users of novel technologies, recognizing how their unique prior experiences and habitual ways of thinking, acting, noticing, and feeling equips them with valuable resources to actively shape and drive the use of technologies. The same goes for the farmers’ experiences with the adaptation of practices following the introduction of a robot on a farm. These can be used to identify best practice approaches, which can feed a community of field-robot-using farmers. An example is the strategy of planting flowers on uneven parts of a field which the robot cannot access. Agricultural extension services should pick up on this and develop formats to support farmers in the decision-making and adaptation processes required after the purchase of a field robot. Gaining detailed insight into how farmers react flexibly to changing conditions and disturbances allows researcher to better understand the plasticity of habits in relation to the interplay of continuity and change.

In addition to studies focusing on technology use, previous research has also analyzed changes in agricultural practices more broadly. In the introduction, we referred, for instance, to approaches based on practice theory and articles defining the stages of agricultural practice change (Prager and Posthumus 2010; Schewe and Stuart 2015; Jakku et al. 2019; Klerkx et al. 2019; Kaiser et al. 2024). Our case study, guided by the transactional theory of learning and its focus on disturbances as drivers for change of habits, allows to gain detailed insight into what others have called the initial stages of change including trigger events (Sutherland et al. 2012; Dumont et al. 2025). We have shown how disturbances can, but do not have to be, an occasion for considering habit change. Our findings reveal instances where farmers started an inquiry and concluded to retain the original habit or to come back to the inquiry again when external conditions have changed (e.g., when there is an option for robotic weed control by a contractor). Such observations help to gain nuanced, in-depth understanding of the ambivalences, different temporalities (e.g., an ongoing inquiry and a later return to it) and non-linearity of potential habit change processes. For a farmer, noticing that weed control robots had become available was disruptive, given their existing habits, such as an interest in robotics. Here, the disturbance refers to newly encountered information, but also an excitement of seeing a field robot. Other disturbances relate to hard, physical work of weeding while removing remaining weeds and thereby relying on bodily experiences. Also observing how the robot stopped on the field because it could not drive on a hill disturbed a habit.^2^ The identification of these different disturbances of habits helps to pinpoint the moments in farmers’ everyday life, in which change comes about and, as such, to gain insight into the making of change in and through action.

Similar to the other approaches (Prager and Posthumus 2010; Chantre and Cardona 2014; Schewe and Stuart 2015), transactional theory of learning considers both intrapersonal aspects (values, emotions, knowledge) and more structural aspects. In the conducted analysis of farmers’ disturbances and inquiries, the focus lays on their interplay, so that the entanglements of intrapersonal aspects, such as previous experiences with technologies or excitement for robotics, and more structural ones like the availability of field robots become visible. The detailed analysis of inquiries (and potential phases such as pinpointing problems, developing working hypotheses, and testing solutions) allows to consider the different aspects that were found to be relevant without assuming (in advance) their relevance for a particular habit. We argue that in this regard analyzing habits, defined as more fine-grained than practices, helps in precisely understanding the change processes in the interplay of actor’s prior experiences (intrapersonal) and more structural aspects.

Several habits co-constitute broader practices, such as the organic cultivation of sugar beet. Through the analytical perspective of transactional theory of learning, we describe habit changes such as the new habits of simultaneous manual hoeing and robot supervision. These habit changes introduced broader practice change in some instances. For the two farmers who established habits of robotic weed control, this was the starting point for the practice of growing organic sugar beet in the first place. For habits of other farmers, such as the farmer who concluded that he prefers robotized weed control by a contractor (habitual belief), it remained unclear to what extent the habit change will affect practices. This brings us to the limitation of the presented case study and empirical approach.

### Limitations and needs for future research

The research presented has some limitations, which we summarize and, on this basis, formulate guidelines for further research. A limitation of our case study is the sample size (see also “Case study design”), which, however, we also relativize with regard to the size of the population of our object of study. We relied mainly on interview data. This type of data allowed us to see certain aspects, in particular consideration processes, while we could not observe other aspects such as disturbances in situ. Future research should dedicate to this, and develop research designs for investigating the continuity and change of habits and practices related to agricultural technology use.

We conduct an in-depth, ‘high resolution’ analyses of particular practices. A trade-off of this micro-lens perspective is the loss of the wide-angle view. Future studies need to conduct in-depths analyses across a variety of settings and contexts to move beyond the particularity of single cases and to identify patterns to arrive at more generalized knowledge about how farmers change their practices. It would move beyond the scope of one paper and requires a more broadly shared collective research effort to do so. We hope the presented approach and its application can contribute to facilitating this.

In connection with this, we also point out that changes in habits can not to be equated with changes in practices, which in turn do not necessarily result in socio-technical change. We focus on habits (very fine-grained, micro practices) to gain insight into practices in the context of attempts to achieve macro-level socio-technical sustainability transitions. This approach is based on theoretical assumptions and arguments on the relevance of habits for practices (Van Poeck and Östman 2021, p. 157) and practices for change and transition dynamics (Shove and Walker 2010). However, the relation between habits and practices and broader societal transitions requires further attention and investigation. Other approaches (e.g., historical, retrospective studies about past transitions) are better suited to gain insight into long-term processes of profound societal transformation. Trade-off there is that they lack detailed insight into the making of it. Our article focuses on the latter, the makings of habit and practice change. This is another way in which our analysis goes into depth, rather than looking at phenomena from a wider angle. By focusing on observable practices here and now, we aim at creating actionable knowledge to facilitate processes of practice change and changes in socio-technical systems. This requires a detailed understanding of how habit and practice change occur in action, something that eludes the wide-angle view and retrospective lens. Prospectively or in the present, we cannot pinpoint the impact of what happens today on potential long-term societal transitions as these are complex and open-ended processes (Köhler et al. 2019).

## Data Availability

The transcripts of the qualitative interviews cannot be published to ensure the interviewees anonymity.

## Appendix 1: Conducted qualitative semi-structured interviews

**Table:**

Abbreviation	Interviews	Date
I1	Cultivation expert and consultant in the region of northeastern Germany	25.10.23
I2	Farmer using the FD robot	07.11.23
I3	Representative from a local business development office	13.11.23
I4	Two farmers currently not cultivating sugar beets	23.11.23
I5	Farmer using the FD robot	08.12.23
I6	Farmer currently not cultivating sugar beets	13.12.23

## Appendix 2: Field notes from attended meetings and events

**Table:**

Abbreviation	Data type	Date
P1	Protocol from project meeting with agronomists, social scientists, technology developers, and a representative from the local sugar processing company (in the following protocol from project meeting)	11.08.23
P2	Protocol from project meeting	08.09.23
P3	Protocol from project meeting	11.10.23
P4	Protocol from project meeting	21.11.23
P5	Protocol from project meeting	10.01.23
P6	Protocol from project meeting	07.02.23
P7	Protocol from project meeting	20.03.23
P8	Protocol from project meeting	10.04.23
P9	Protocol from project meeting; in addition to the above-mentioned actors, a farmer was present	25.04.23
P10	Protocol from project meeting	06.06.24
FN1	Field notes from a field day with presentations of weed control technologies (and in particular field robots)	19.06.2024
P11	Protocol from project meeting; in addition to the above-mentioned actors, a farmer was present	05.07.24

## Footnotes

1 Names of farmers changed for anonymity.

2 For a similar categorization of bodily, intellectual, and observative disturbances, see Östman et al. (2019).
